# Supplementary material for: AffyMAPSDetector: a software tool to characterize Affymetrix GeneChip™ expression arrays with respect to SNPs
Source: BMC Bioinformatics. 2007 Jul 30;8:276. doi: 10.1186/1471-2105-8-276 (PMC1959249; doi:10.1186/1471-2105-8-276)
Supplement: Additional file 13 — AffyMAPSDetector v1 source code. AffyMAPSDetector source code. [file 1471-2105-8-276-S13.zip › AffyMAPSDetector_v1_src/doc/overview-tree.html]

Class Hierarchy


|  |  |  |  |
| --- | --- | --- | --- |
| **Overview** \| Package \| Class \| Use \| **Tree** \| **Deprecated** \| **Index** \| **Help** | | |  |
| PREV   NEXT | **FRAMES**    **NO FRAMES**     **All Classes** |  |  |


---


## Hierarchy For All Packages

**Package Hierarchies:**: affychip.snpdetector, affychip.snpdetector.model, affychip.snpdetector.model.event, affychip.snpdetector.resources, affychip.snpdetector.ui, affychip.snpdetector.util, jwf

---

## Class Hierarchy

- class java.lang.Object
  - class affychip.snpdetector.**AffyMapsDetectorLauncher**- class affychip.snpdetector.resources.**AppIcons**- class affychip.snpdetector.util.**ColorGradient**- class java.awt.Component (implements java.awt.image.ImageObserver, java.awt.MenuContainer, java.io.Serializable)
          - class java.awt.Container
            - class javax.swing.JComponent (implements java.io.Serializable)
              - class javax.swing.JInternalFrame (implements javax.accessibility.Accessible, javax.swing.RootPaneContainer, javax.swing.WindowConstants)
                - class affychip.snpdetector.ui.**AffyMapsDetectorFrame** (implements affychip.snpdetector.model.event.StatusEventListener)- class affychip.snpdetector.ui.**CrossHbCheckFrame**
                - class javax.swing.JPanel (implements javax.accessibility.Accessible)
                  - class jwf.**Wizard** (implements java.awt.event.ActionListener)- class jwf.**WizardPanel**
                      - class jwf.**NullWizardPanel**- class affychip.snpdetector.ui.**StepOnePanel**- class affychip.snpdetector.ui.**StepThreePanel**- class affychip.snpdetector.ui.**StepTwoPanel**
              - class java.awt.Window (implements javax.accessibility.Accessible)
                - class java.awt.Dialog
                  - class javax.swing.JDialog (implements javax.accessibility.Accessible, javax.swing.RootPaneContainer, javax.swing.WindowConstants)
                    - class jwf.**ErrorMessageBox** (implements java.awt.event.ActionListener)- class affychip.snpdetector.ui.**FileLoaderWizardDialog** (implements jwf.WizardListener)- class affychip.snpdetector.util.**GeneAnnotLoaderWizard** (implements jwf.WizardListener)
                  - class java.awt.Frame (implements java.awt.MenuContainer)
                    - class javax.swing.JFrame (implements javax.accessibility.Accessible, javax.swing.RootPaneContainer, javax.swing.WindowConstants)
                      - class affychip.snpdetector.ui.**AffyMapsDetectorUI**- class affychip.snpdetector.util.**ComplementDnaSeq**- class affychip.snpdetector.util.**DuplicateRemover**- class affychip.snpdetector.util.**PmMmHeatMapViewer**
          - class affychip.snpdetector.**Constant**- class affychip.snpdetector.model.**DataState** (implements java.io.Serializable)- class java.util.EventObject (implements java.io.Serializable)
                - class affychip.snpdetector.model.event.**StatusEvent**
                - class affychip.snpdetector.model.**GeneInfo**- class affychip.snpdetector.util.**GuiUtils**- class affychip.snpdetector.util.**PmMmHeatmapViewerModel**- class affychip.snpdetector.util.**PmMmHeatmapViewerModel.Row** (implements java.lang.Comparable)- class affychip.snpdetector.util.**PmMmHeatmapViewerModel.RowComparatorForDataSorting** (implements java.util.Comparator)- class affychip.snpdetector.util.**PmMmMatrixLoader**- class affychip.snpdetector.model.**Probe** (implements java.lang.Comparable)- class affychip.snpdetector.model.**ProbeSet** (implements java.lang.Comparable)- class affychip.snpdetector.resources.**ResourceLoader**- class affychip.snpdetector.model.**SnpDetector**
                                    - class affychip.snpdetector.model.**OptimizedSnpDetector**
                                    - class affychip.snpdetector.model.**SnpFinder**- class affychip.snpdetector.model.**SnpInfo**- class affychip.snpdetector.model.**SnpMatcher**- class java.lang.Thread (implements java.lang.Runnable)
                                            - class affychip.snpdetector.model.**SnpMatcher.FileClosingShutdownHook**
                                            - class jwf.**WizardAdapter** (implements jwf.WizardListener)- class jwf.**WizardContext**- class jwf.**WizardUtils**

## Interface Hierarchy

- interface java.util.EventListener
  - interface affychip.snpdetector.model.event.**StatusEventListener**
  - interface jwf.**WizardListener**

---


|  |  |  |  |
| --- | --- | --- | --- |
| **Overview** \| Package \| Class \| Use \| **Tree** \| **Deprecated** \| **Index** \| **Help** | | |  |
| PREV   NEXT | **FRAMES**    **NO FRAMES**     **All Classes** |  |  |


---
